# Classifying Alcohol Control Policies with Respect to Expected Changes in Consumption and Alcohol-Attributable Harm: The Example of Lithuania, 2000–2019

**DOI:** 10.3390/ijerph18052419

**Published:** 2021-03-02

**Authors:** Jürgen Rehm, Mindaugas Štelemėkas, Carina Ferreira-Borges, Huan Jiang, Shannon Lange, Maria Neufeld, Robin Room, Sally Casswell, Alexander Tran, Jakob Manthey

**Affiliations:** 1 Institute for Mental Health Policy Research, Centre for Addiction and Mental Health (CAMH), 33 Ursula Franklin Street, Toronto, ON M5S 2S1, Canada; jtrehm@gmail.com (J.R.); hedy.jiang@utoronto.ca (H.J.); shannon.lange@camh.ca (S.L.); neufeld.maria@gmail.com (M.N.); 9trana@gmail.com (A.T.); 2 Campbell Family Mental Health Research Institute, CAMH, 250 College Street, Toronto, ON M5T 1R8, Canada; 3 Center for Clinical Epidemiology and Longitudinal Studies, Institute of Clinical Psychology and Psychotherapy, Technische Universität Dresden, Chemnitzer Str. 46, 01187 Dresden, Germany; 4 Center for Interdisciplinary Addiction Research (ZIS), Department of Psychiatry and Psychotherapy, University Medical Center Hamburg-Eppendorf (UKE), Martinistraße 52, 20246 Hamburg, Germany; 5 Department of Psychiatry, University of Toronto, 250 College Street, 8th Floor, Toronto, ON M5T 1R8, Canada; 6 Dalla Lana School of Public Health, University of Toronto, 155 College Street, 6th Floor, Toronto, ON M5T 3M7, Canada; 7 Institute of Medical Science (IMS), University of Toronto, Medical Sciences Building, 1 King’s College Circle, Room 2374, Toronto, ON M5S 1A8, Canada; 8 Department of International Health Projects, Institute for Leadership and Health Management, I.M. Sechenov First Moscow State Medical University, Trubetskaya Str., 8, b. 2, 119992 Moscow, Russia; 9 Health Research Institute, Faculty of Public Health, Lithuanian University of Health Sciences, Tilžės Str. 18, 47181 Kaunas, Lithuania; mindaugas.stelemekas@gmail.com; 10 Department of Preventive Medicine, Faculty of Public Health, Lithuanian University of Health Sciences, Tilžės Str. 18, 47181 Kaunas, Lithuania; 11 WHO European Office for Prevention and Control of Noncommunicable Diseases, Leontyevsky Pereulok 9, 125009 Moscow, Russia; ferreiraborgesc@who.int; 12 Centre for Alcohol Policy Research, Building NR-1, La Trobe University, Plenty Rd. x Kingsbury Rd., Bundoora, VIC 3086, Australia; r.room@latrobe.edu.au; 13 Centre for Social Research on Alcohol and Drugs, Department of Public Health Sciences, Stockholm University, 3rd Floor, Sveavägen 160, 113 46 Stockholm, Sweden; 14 SHORE and Whariki Research Centre, College of Health, Massey University, 90 Symonds Street, Auckland 1010, New Zealand; S.Casswell@massey.ac.nz; 15 Department of Psychiatry, Medical Faculty, University of Leipzig, Semmelweisstraße 10, 04103 Leipzig, Germany

**Keywords:** alcohol control policy, best buys, classification, evaluation, taxation, availability, marketing, Lithuania

## Abstract

Due to the high levels of alcohol use, alcohol-attributable mortality and burden of disease, and detrimental drinking patterns, Lithuania implemented a series of alcohol control policies within a relatively short period of time, between 2008 and 2019. Based on their expected impact on alcohol consumption and alcohol-attributable harm, as well as their target population, these policies have been classified using a set of objective criteria and expert opinion. The classification criteria included: positive vs. negative outcomes, mainly immediate vs. delayed outcomes, and general population vs. specific group outcomes. The judgement of the alcohol policy experts converged on the objective criteria, and, as a result, two tiers of intervention were identified: Tier 1—highly effective general population interventions with an anticipated immediate impact; Tier 2—other interventions aimed at the general population. In addition, interventions directed at specific populations were identified. This adaptable methodological approach to alcohol control policy classification is intended to provide guidance and support for the evaluation of alcohol policies elsewhere, to lay the foundation for the critical assessment of the policies to improve health and increase life expectancy, and to reduce crime and violence.

## 1. Introduction

Lithuania is a Baltic country located in the northeast of the European Union. It regained independence in 1990 after being part of the Soviet Union for approximately 50 years. Although its drinking culture was somewhat influenced during that period by Soviet drinking patterns, Lithuania has had a long cultural history of its own [1]. We start the discussion about alcohol policy after independence, when a back-and-forth between economic and public health interests took place and ultimately led to relatively marked changes in alcohol consumption trends for the country (see Appendix A Table A1). Since 1993, the year with the lowest level of alcohol consumption, alcohol consumption in Lithuania has more than doubled, starting at 6.2 liters of pure alcohol per capita and reaching its peak in 2011/12 at more than 15 liters per capita (see Appendix A Table A1; [2,3]). Similarly, the proportion of current drinkers in the adult population also increased considerably, from 55% in 1993 to 80% in 2016 [2]. Accordingly, alcohol-attributable mortality in Lithuania was among the highest in 2016 globally, with an estimated 9894 deaths, or 24.2% of all deaths [4], despite experiencing a decline in consumption after 2008 when several alcohol control policy measures associated with a reduction in the level of alcohol consumption were implemented.

However, it is difficult to assess the effects of the policies implemented in 2008 as they cannot be easily distinguished from the effects of the global financial crisis of 2008/2009 [5], and abrupt changes to Lithuanian non-alcohol taxation policies (e.g., an introduction of a formal requirement to pay health insurance taxes) during the same time period [6]. These two economic events resulted in a steep increase in the number of declared emigrations from Lithuania in 2010, and thus in a reduction in its official population size (and given the same number of deaths in the country, in an increase in mortality rates).

Between 1 January 2016 and 1 January 2018, Lithuania once again began implementing a number of far-reaching alcohol control policy interventions, including all three of the World Health Organization’s (WHO) “best buys” for alcohol (an increase in prices for alcoholic beverages due to taxation, a reduction in the availability of alcoholic beverages, and a ban on advertisement of alcohol [7,8]). According to the WHO, a “best buy” is defined as an intervention for which there is compelling evidence that it is not only highly cost-effective, but also feasible, low in cost, and appropriate to implement within the constraints of the local healthcare system [9]. Before continuing further, we would like to refer to the overview authored by Miščikienė and colleagues [10], which not only lists the individual alcohol control policy interventions, but also gives more context to the historical processes involved in policy making in Lithuania over the past 30 years. The current publication will restrict itself to the time period spanning the past 20 years, a time period which covers all the major interventions implemented in Lithuania to control the use of alcohol.

Evaluating the impact of alcohol control policy implementation in Lithuania has the advantage of allowing us to make generalizations about the enactment of alcohol policies in other high-income countries, as it is a high-income country with a stable democracy, is part of the European Union, and is integrated into the North Atlantic Treaty Organization. It is also a country with a population of less than 3 million with strong surveillance systems in place that monitor alcohol consumption and burden of disease in great detail, allowing for comprehensive analyses. Thus, Lithuania offers a unique opportunity to simultaneously evaluate the impact of the three “best buys”, all implemented within a short time span, on a wide range of outcomes (e.g., total adult alcohol per capita consumption, alcohol-attributable hospitalizations, mortality, and violence). Over the course of 12 years, beginning in 2008, Lithuanian policy-makers voted in favor of over a dozen alcohol policies, affecting very different aspects of alcohol use. Prior to evaluating these policies, each must be critically reviewed with regard to their expected effects and possible confounding of their effects with other policies or economic trends. In the following, we undertake this assessment and classify the interventions into two main tiers, ordered by the degree of impact expected, and report on a sensitivity analysis of this ordering using a different methodology. In addition to the policies included in these two tiers, which impacted the whole population, there were also alcohol policy changes which targeted specific groups within the population. These will not be discussed in detail here.

The present contribution aims to present a hierarchical classification of the respective policies according to their expected effects. Although research in this area is increasing and various alcohol policy-scoring tools have been developed (e.g., [11,12,13,14]), to our knowledge, this paper represents the first attempt to develop and apply a formalized approach to classifying alcohol policies prior to assessing the impact of these policies on a variety of outcomes in greater detail.

## 2. Materials and Methods

### 2.1. General Design of Alcohol Control Policy Evaluations

Since the effect of policy changes cannot be tested through traditional randomized controlled trial designs, well-selected, -designed, and -analyzed natural experiments are the method of choice for examining the impact of policy changes on outcomes [15,16,17]. The classic methodology for evaluation is interrupted time series analysis, where the observed trend is compared to the expected trend, the latter based on data collected prior to the intervention [18]. Obviously, as these are not randomized experiments, but essentially before-and-after comparisons, the more relevant control variables that are included, the better [19], though there are limitations due to sample size and statistical power, and there is always the possibility of overfitting regression models [20]. Most of these potential confounders will be economic variables, as short- and medium-term changes at the level of alcohol consumption have been shown to depend to a large degree on economic factors [2,21,22,23]. Thus, statistically controlling for important confounders such as inflation, unemployment, or gross domestic product will help rule out alternative explanations.

For any interrupted time series analysis, the nature of the effect needs to be specified a priori (e.g., abrupt effects, which are usually expected to start immediately after implementation and are expected to have a lasting impact, except for financial diminution due to inflation; or lagged effects, where only part of the full effect is expected immediately after the intervention is enacted, and the full effect may only be realized after some time has passed, possibly several years [24]). Given this methodology, it is necessary that a sufficient number of data points are available for the period prior to the implementation of the first big alcohol policy intervention before it is analyzed (for Lithuania: January 2008), and that there is also a sufficient number of data points after the last intervention was enacted for it to be modeled. For interrupted time series analyses, a minimum of 50–100 time points in total would normally be required, with at least 24 before and 24 after the intervention was implemented ([18]; see also [25]). We can therefore only use time series methodology for monthly data (yearly data would not provide a sufficient number of time points). In order to achieve the minimum number of time points, we scored policies within a time series between January 2000 and December 2019. Although there were a number of policies introduced during this period, we could not appropriately score the earliest policies which reduced harm (i.e., June 2001) as they did not contain a sufficient number of time points to analyze, and neither did we score any policies that one would expect would actually increase alcohol-attributable harm (e.g., policies that increased alcohol availability).

Our time series dataset, therefore, provided us with 72 months of data prior to the enactment of the first major alcohol control policy which was expected to reduce alcohol-attributable harm by 2008. Any intervention introduced after 1 January 2018 would likely run into power issues given that the data would likely not yet be available. Such considerations are relevant to any potential interrupted time series evaluations of alcohol control policies.

### 2.2. Dimensions of Alcohol Control Policies Relevant for Evaluating Impact

In order to establish hypotheses about alcohol control policies, we applied objective classifications and expert judgement. For the objective classifications, the following dichotomies were deemed to be relevant (based on [26,27]):

- Positive vs. negative outcomes: Policies which are known to increase harm, most often by increasing the level of consumption, vs. policies which are known to decrease harm. For instance, considering policies which were implemented in Lithuania, restrictions on availability (e.g., by reducing opening hours) can be expected to decrease use and harm [28,29]. On the other hand, widening availability, for example, by liberalizing alcohol sales and production or allowing sales of alcohol in petrol stations, are postulated to increase use and harm ([30]);
- Mainly immediate vs. delayed outcomes: Policies where much of the impact on use or harm is immediate (e.g., availability, price increases via taxation) vs. policies where the impact is more medium- or long-term (ban on advertisements, education on alcohol-attributable harm).
- General population vs. specific group outcomes: Policies also differ with respect to their population reach: an increase in alcohol excise taxation applies equally to everyone in a country, even though only drinkers and their families will likely be most strongly affected. On the other hand, a change in the minimum drinking age mainly applies to alcohol use by people in this age group, and to the resulting attributable harm to them. Similarly, a law mandating an ignition interlock device for drivers convicted for driving under the influence of alcohol would apply only to drivers with convictions for driving under the influence. For the latter policy, we would mainly expect consequences to the number of traffic injuries, whereas successfully implemented alcohol policies with a general reach should impact many social and health outcomes, including traffic injuries [31].

### 2.3. Main Selection Criteria

#### 2.3.1. Tier 1: Highly Effective General Population Interventions with an Anticipated Immediate Impact

We based our selection of the most impactful interventions on prior empirical evaluations by the WHO [7,8,32] and independent study groups (e.g., [27]). Furthermore, we selected interventions where the main impact of the effects on alcohol use was predicted to begin immediately (such as pricing and availability policies), allowing for an estimation of effects via interrupted time series methodology [13]. Thus, a ban on marketing alone would be excluded from Tier 1 interventions, as effects would only accumulate over time [27].

We decided to operationalize pricing interventions as a rise in the excise taxation of alcohol, which would be associated with a decrease in affordability in the 12 months following enactment, so that it would be included only when the price of alcohol increased at a higher rate than the average disposable income (see also [33]). This definition was operationalized according to the definitions of Statistics Lithuania [34], which publishes a yearly index for the price of alcoholic beverages, and an index for average disposable income per month, per household member. The affordability index was then constructed as follows:

Proportional changes in the affordability index, from year t to year t + 1: ((Income_t+1_/alcohol price_t+1_) − (Income_t_/alcohol price_t_))/(Income_t_/alcohol price_t_)

Thus, if disposable income increased more than the price (relative to the previous year), the affordability index would be positive (higher affordability), and if the opposite were true, the affordability index would be negative.

For Tier 1 interventions which involved taxation and thus price, we only included interventions which were associated with a reduction in affordability (i.e., negative changes in the affordability index in Table 1). For availability, we included interventions aimed at reducing alcohol use for large parts of the general population, for instance, by restricting hours of sale for alcoholic beverages, and which had shown similar effect sizes in prior research (e.g., [29]). Thus, not all availability restrictions and taxation increases qualified for inclusion in Tier 1. Moreover, any intervention which targeted only specific groups was excluded from Tier 1 (and Tier 2).

#### 2.3.2. Tier 2: Other Interventions Aimed at the General Population

For Tier 2, as well as all interventions in Tier 1, we included taxation or price increases up to a change in affordability of 5%, which was the median for the affordability change index. We also classified general availability restrictions as Tier 2 interventions, which were expected to produce smaller effects, such as increased restrictions which were limited to only certain days (see also [29]).

#### 2.3.3. Alcohol Control Interventions Not Aimed at the General Population

A number of alcohol control interventions were designed to target specific groups or situations and not the general population. These interventions will be used to test specific hypotheses (e.g., regarding drink-driving interventions impacting on traffic crashes, traffic injuries, and traffic fatalities; [35]).

### 2.4. Sensitivity Analysis

For the sensitivity analysis, interventions were independently established by a panel of alcohol policy experts using a modified nominal group technique [36], whereby experts gave individual judgments without an interactive group meeting. All alcohol control policy measures during the time period 2000–2019 (see Table 2) were rated. Five alcohol control policy experts were selected to provide the ratings, none of whom at the time of their rating were familiar with the specific data for Lithuania at the level of alcohol consumption, the mortality and other disease burden data, or the data on affordability in Lithuania: Sally Casswell, Carina Ferreira-Borges, Shannon Lange, Maria Neufeld, and Robin Room. The ratings were conducted in May of 2020.

Using this procedure, the experts could not be biased by knowledge of the actual Lithuanian history of associations between policies and mortality. The rating scale applied for each policy ranged from 0 to 10, and ratings were based on the perceived immediate impact of alcohol use on health, based on the following instructions: “Please rank the alcohol policy measures in their predicted immediate impact on alcohol consumption and health (think about all-cause mortality as the main health outcome) from 0 to 10 (0 = no impact to 10 = highest impact)”.

Interrater reliability, as measured by the intraclass correlation coefficient, was 0.54, with a 95% CI between 0.35 and 0.72, and a median of 0.71. An interquartile range of 0.18 for the Spearman rank correlations between raters indicated fair to good agreement [37].

Dates were then selected as being important for modeling purposes if at least one policy implemented during a given time period had an average rating of 5 or more (see highlighted rows in Table 2).

## 3. Results

### 3.1. Tier 1 Alcohol Control Interventions: Highest Impact on General Population Expected

Based on the strict criteria used, only three policy enactments qualified between 2000 and 2019 for inclusion: 1 January 2008, 1 March 2017, and 1 January 2018 (further details provided in Table 2; see also [10,35]).

On 1 January 2008, the Lithuanian parliament declared the “Year of Sobriety” and increased excise tax by 20% for ethyl alcohol, wine, and intermediate products, and by 10% for beer. Furthermore, and not strictly related to Tier 1 interventions but enacted in parallel, drink-driving legislation was toughened by introducing higher penalties, car confiscation, or driver imprisonment for repeat offenders, and a reduction in the blood alcohol concentration (BAC) threshold for young drivers (those having had a driving license for less than two years) from 0.4 to 0.2 per mille. Lastly, alcohol advertising was restricted during the daytime for TV and radio, with an indication that there would be a full advertising ban in a few years [10,38]. While we cannot rule out that these additional measures may have confounded the effects of tax increases, this is relatively unlikely, as drink-driving legislation and regulation on advertisement follow other more specific and less immediate pathways to reducing alcohol harm. The global economic crisis hit Lithuania only at the end of 2008 and therefore did not confound the implemented measures (according to economic data based on GDP, the impact was after October 2008; based on [39]).

On 1 March 2017, the highest ever increase in Lithuanian excise tax came into effect during a stable period of gradual economic growth: excise taxes for beer and wine increased by 111–112%, by 91–94% for intermediate products, and by 23% for ethyl alcohol. The only other measure implemented in 2017, on 1 January, was the criminalization of drink-driving for heavily drunk persons. However, the initial version of the amendment contained a significant flaw (a refusal to undergo BAC testing when suspected of driving under the influence was not an offence under Lithuanian law and therefore police could not force someone to comply) [10], in addition to it targeting only a specific and narrow population group; thus, it is quite unlikely that it had a strong initial effect on the general population in that year.

On 1 January 2018, several strong restrictions targeting availability and marketing came into effect. The minimum legal purchase age was increased from 18 to 20 years old, off-premise sales hours were reduced by two hours (becoming 10 a.m. to 8 p.m.), and even further reduced on Sundays (becoming 10 a.m. to 3 p.m.). A full alcohol advertising ban (including TV, radio, and internet) came into effect, with only small exceptions [10]. As with the 2008 alcohol policy measures, a confounding of availability and marketing measures cannot entirely be ruled out but appears unlikely to be present in the short term.

A summary of Tier 1 interventions is given in Box 1.

Box 1 **Tier 1 interventions.**

1 January 2008 Criterion met: affordability(i) drink-driving (increased penalties) (ii) marketing/advertising (banned on TV and radio during daytime) (iii) taxation/price (increase in excise tax by 10–20%)1 March 2017 Criterion met: affordabilitytaxation/price (increase in excise tax: 111–112% for wines and beer; and 23% for ethyl alcohol)1 January 2018 Criterion met: availability(i) availability (increase in legal minimum age and in enforcement; and reduced off-premise sales hours) (ii) marketing/advertisement ban (full ban on TV, radio, and internet advertisements with very few exceptions)

### 3.2. Tier 2 Interventions: Sizable Impact on Alcohol Use and Attributable Harm in the General Population

Based on the definitions provided above, all Tier 1 interventions automatically qualify as Tier 2 interventions (see Box 2). In addition, based on the criteria above, two further policy enactment dates qualify: 1 January 2009 and 1 April 2014.

Box 2 **Tier 2 interventions (two additional interventions in addition to Tier 1 are highlighted in blue).**

1 January 2008(i) drink-driving (increase in penalties) (ii) marketing/advertising (ban on TV and radio during daytime hours) (iii) taxation/price (increase in excise tax of 10–20%)1 January 2009 New; criteria: affordability and availability(i) taxation/price (increase in excise tax of 10–15%; removal of tax exemptions for small beer breweries; and relative price of alcohol increases due to global economic crisis) (ii) availability (off-premise sales restricted at night; and a ban on having opened alcohol beverages in cars)1 April 2014 New; criterion: affordabilitytaxation/price (increase in excise tax by of 10–47% for beer, wine and intermediate products; and 1% for ethyl alcohol)1 March 2017taxation/price (increase in excise tax: 111–112% for wines and beer; and 23% for ethyl alcohol)1 January 2018(i) availability (increase in legal minimum age and in enforcement; and reduced off-premise sales hours) (ii) marketing/advertisement (full ban on TV, radio, and internet advertisements with few exceptions)

Depending on the research question and statistical methodology selected [18], Tier 1 and Tier 2 interventions will be summed to obtain a cumulative score for alcohol control policies as follows: change from 0 to +1 for 1 January 2008; from 1 to 1.5 for January 2009; from 1.5 to 2 on 1 April 2014; from 2 to 3 on 1 March 2017; from 3 to 4 for January 2018.

### 3.3. Sensitivity Analyses: Interventions Based on Expert Judgments

The alcohol control policy interventions selected by the experts are highlighted in Table 2 in light blue. The experts selected all of the Tier 1 and Tier 2 interventions plus two others (from 1 March 2015 and 1 January 2016). In other words, the expert judgments highly corresponded with the Tier 1 and Tier 2 interventions selected by objective criteria. In some ways, the expert judgements could be seen as simply having a lower threshold, albeit one without underlying objective data.

### 3.4. Interventions for Specific Populations

There are also interventions for specific populations such as those targeting a particular age group (e.g., minimum legal drinking age increase from 18 to 20 years) or for traffic participants (e.g., drunk-driving laws). While such measures, under certain circumstances, may impact the overall drinking level of the population [40], these interventions will mainly be modeled for the specific groups or outcomes intended. For example, the effects of increasing the legal drinking age should be seen among the 18- to 20-year-old age group (i.e., those affected by the policy change), and the drunk-driving laws should mainly affect traffic participation and associated outcomes, such as traffic crashes, injuries, or fatalities [31].

## 4. Discussion

With the considerations spelled out above, we were able to identify the alcohol control policies where we would expect the highest overall impact. The subjective classifications of the various policies by the panel of alcohol policy experts converged with the classification of policies using objective criteria alone. Before we discuss these results further, we would like to point out their limitations.

First, not a lot of research exists on the comparative effectiveness of differing methods used in implementing the best buys. For instance, increases in excise taxation or setting a minimum unit price in many categorization systems are seen as a “best buy”, even if the taxation increase does not even cover inflation and/or increases in disposable income, or the minimum price is so low that it would not affect consumer behavior. While pricing policies clearly are potentially effective and cost-effective, their overall effectiveness will depend on the overall economic situation and on the affordability of the alcoholic beverages affected. In addition, definitions of affordability vary [41,42], as do underlying data sources. We used a simple definition based on disposable income and the average price of alcoholic beverages. There are other definitions which would take into account the consumer price index as well, as this would reflect the price of alternative goods which could be obtained [42]. Future research will have to decide which affordability index is best correlated with actual behavior, and thus with consumption and alcohol-attributable harm. For interventions on availability, there is much less coherent theory and research available which quantitatively compare the impact of different forms of availability (for general considerations: [27,43]). As a result, the classification of interventions into Tier 1 and Tier 2 categories based on availability is on less solid ground. Finally, while the impact of marketing and advertising on consumption is well established [44], the impact of policies to control these is much less clear (e.g., [27,45,46]). As a consequence, even the classification system used to gauge the impact of marketing and advertisement bans, such as the one used for Lithuania, is problematic. Finally, the interrater reliability ratings of the experts were relatively low, suggesting a need for further research on the methods used to evaluate alcohol control policy measures.

While many attempts have been made to classify and evaluate the potential impact of alcohol control policies (e.g., [26,27]), fewer attempts have been made to quantify in detail the potential impact based on objective criteria for a particular country in order to provide the groundwork for formal evaluations. Furthermore, current alcohol policy impact scales are usually based on expert ratings, with only a few studies available to date which test if changes in policy scores were in fact related to changes in consumption or attributable harm. We clearly need more impact studies, and more rigorous ones, which assess both single and combined interventions to allow us to make better recommendations on which policies are best to implement. Further, these studies should have more important public health endpoints. For instance, only one of the 50 studies in the meta-analyses on taxation and health outcomes by Wagenaar and colleagues [47] examined all-cause mortality, and none examined life expectancy. However, these are the indicators politicians are interested in, as a recent case study on alcohol policy from Russia amply demonstrated, when such outcomes were taken up by many media outlets and discussed at the UN and in several parliaments [48].

## 5. Conclusions

The classification procedure, as described, resulted in the identification of three alcohol policy categories, based not only on experts’ opinions, but also on objective criteria which were transparently described and based on available evidence. The resulting classification system allows us to clearly distinguish between expected effects of alcohol control policies and potential confounding factors, such as other policies not related to alcohol regulation or economic trends.

Monitoring and evaluating progress in alcohol policy implementation and identifying policies with the highest impact on a population’s health and economic prosperity are key components when implementing health strategies and interventions [49]. The detailed methodological approach to alcohol policy classification within a country, as described here, can inform monitoring and evaluation approaches for other countries and can be adapted to different situations based on available data.

Importantly, the present contribution lays the foundation for us to critically assess the potential for specific alcohol control policies to improve health and increase life expectancy, as well as to reduce crime and violence. The formal evaluation which follows will provide important information regarding the value of the chosen approach, especially as it compares with various expert scales—which, to date, have not shown a high association with actual levels of drinking or alcohol-attributable harm.

## Figures and Tables

**Table 1:** Income, alcohol prices, and changes in the affordability index over the period of alcohol policy interventions in Lithuania.

Year	Average Disposable Income Per Month Index, Per Household Member 2010 = 100	Alcohol Price Index (API) 2010 = 100	Affordability Index (Defined as Disposable Income/API) 2010 = 100	Proportional Changes in Affordability Index (Compared to Previous Year)
2007	92.4	80.4	115	14.4%
2008	103.9	90.6	115	−0.1%
2009	116.8	98.9	118	3.0%
2010	100.0	100.0	100	−15.3%
2011	88.1	98.9	89	−10.9%
2012	97.1	101.3	96	7.6%
2013	106.4	102.5	104	8.3%
2014	112.3	104.1	108	3.9%
2015	123.7	105.0	118	9.1%
2016	133.0	107.3	124	5.3%
2017	138.6	119.9	116	−6.7%
2018	149.1	122.4	122	5.3%
2019	160.3	124.9	128	5.4%

**Table 2:** Major alcohol control policies implemented between 2001 and 2019 in Lithuania (for further details, see [10,35]).

#	Date of Policy Implementation	Type of Policy	Policy Score	Expected Impact
1	1 June 2001	taxation/price (increase in excise tax of 6% for ethyl alcohol; and change to a taxing format)	0	0
2	28 November 2001	availability (liberalization of sales and production; and sales of alcohol in petrol stations allowed)	0	Negative *
3	28 June 2002	(i) availability (state production monopoly abolished; and municipalities allowed to decide on alcohol sales) (ii) marketing/advertising (liberalization of advertising, fines for violations reduced)	0	Negative *
4	1 July 2002	taxation/price (exemption of excise tax for small breweries)	0	Negative *
5	1 May 2003	Drink-driving (criminal liability restored in certain cases when individuals are harmed, or property is damaged significantly)	0	0
6	16 July 2003	marketing/advertising (liberalization of advertising by expanding the range of display places)	0	Negative *
7	1 January 2004	taxation/price (decrease of excise tax of a sub-category of fermented beverages when equalizing the tax with other similar categories)	0	Negative *
8	1 May 2004	taxation/price (four alcohol beverage categories formed, licencing changes when joining the EU)	0	0
9	1 January 2008	(i) drink-driving (increased penalties) (ii) marketing/advertising (banned on TV and radio during daytime) (iii) taxation/price (increase excise tax by 10–20%)	Tier 1: Affordability ^&^ decrease among others	1
10	1 January 2009	(i) taxation/price (increase excise tax by 10–15%; removal of tax exemptions for small beer breweries; and relative price of alcohol increases due to global economic crisis) (ii) availability (off-premise sales restricted at night; and a ban on having opened alcohol beverages in cars)	Tier 2: Affordability ^&^ < 5%	0.5
11	1 April 2014	taxation/price (increase excise tax by 10–47%; and 1% for ethyl alcohol)	Tier 2: Affordability ^&^ < 5%	0.5
12	1 January 2015	drink-driving (0% BAC for select drivers)	0	Specific measure
13	1 March 2015	taxation/price (increase in excise tax of 10–16% for beer, wine, and intermediate products; and 2% for ethyl alcohol)	0	0
14	1 January 2016	availability (sales banned at petrol stations)	0	Specific measure
15	1 March 2016	taxation/price (increase excise tax by 8% for beer and wine; and 2.5% for ethyl alcohol)	0	0
16	1 January 2017	drink-driving (>0.15% BAC = criminal offence); see #20	0	Specific measure
17	1 March 2017	taxation/price (increase in excise tax of 111–112% for wine, beer, and intermediate products; and 23% for ethyl alcohol)	**Tier 1: Affordability ^&^ descrease**	1
18	1 January 2018	(i) availability (increase in legal minimum age and in enforcement; and reduced off-premise sales hours) (ii) marketing/advertisement (full ban of TV, radio, and internet advertisements with few exceptions)	**Tier 1: availability**	1
19	1 March 2019	taxation/price (10.5% increase for ethyl alcohol)	0	Not to be considered for time series analysis (power)
20	1 April 2019	from 1 January 2017, drink-driving with a BAC level higher than 1.5 permilles was criminalized. However, the original law was flawed, since refusal to be tested by the police did not result in a criminal offence. This was later amended and came into effect on 1 April 2019.	0	Specific measure; not to be considered for time series analysis (power)
21	1 November 2019	“child champagne” ban (prohibited to manufacture and to sell food, toys, and other goods for children and adolescents with designs mimicking alcoholic beverages and/or their packaging).	0	Not to be considered for time series analysis (power)

Note: Affordability ^&^ here was defined as yearly changes in the ratio between real household disposable income and alcohol price index. Higher affordability indicated that alcoholic beverages became more affordable. Negative * here means that the alcohol policy was predicted to lead to an increase in alcohol per capita consumption. Blue-highlighted interventions have been rated by alcohol policy experts as being effective.

## Data Availability

All the data used in this article were from open sources, mainly from the Official Statistics Portal of the Lithuanian Department of Statistics available at https://www.stat.gov.lt/ (accessed on 5 January 2021).

## Appendix A

**Table A1:** Alcohol per capita consumption in Lithuania 1991–2019.

Year	Total Alcohol Per Capita Consumption * [2]	Recorded Alcohol Per Capita Consumption [3]
1990	11.9	
1991	9.9	
1992	8.3	
1993	6.2	
1994	7.3	
1995	9.0	7.9
1996	10.4	7.9
1997	10.4	8.7
1998	10.4	8.0
1999	11.8	8.6
2000	13.9	9.7
2001	15.4	10.5
2002	15.5	11.1
2003	15.8	11.3
2004	15.9	12.2
2005	16.0	12.5
2006	16.0	13.2
2007	15.9	13.9
2008	15.3	13.9
2009	15.0	13.1
2010	15.1	13.5
2011	16.1	14.7
2012	16.5	14.7
2013	16.4	14.5
2014	16.1	14.2
2015	15.5	14.0
2016	15.1	13.2
2017	14.7	12.3
2018	14.7	11.2
2019	14.5	11.1

* Total alcohol per capita consumption comprises recorded, unrecorded, and tourist consumption (for definitions, see [50,51]).
